# What are the motivations and barriers to pre-exposure prophylaxis (PrEP) use among black men who have sex with men aged 18–45 in London? Results from a qualitative study

**DOI:** 10.1136/sextrans-2018-053773

**Published:** 2019-03-04

**Authors:** T Charles Witzel, Will Nutland, Adam Bourne

**Affiliations:** 1 Sigma Research, Department of Public Health, Environments and Society, London School of Hygiene and Tropical Medicine, London, UK; 2 Australian Research Centre for Sex, Health and Society, La Trobe University, Melbourne, Victoria, Australia

**Keywords:** risk, pre-exposure prophylaxis, health status disparities, England, sexual and gender minorities

## Abstract

**Background:**

Black men who have sex with men (BMSM) have higher HIV incidence and prevalence when compared with other men who have sex with men, despite similar risk profiles. New prevention technologies, including pre-exposure prophylaxis (PrEP), may be effective in responding to these inequalities, provided they are appropriately targeted and acceptable to their intended beneficiaries. This study aims to understand the motivations and barriers of BMSM aged 18–45 to PrEP uptake.

**Methods:**

Twenty-five BMSM recruited through geolocation social networking apps took part in in-depth interviews between April and August 2016. Intersectionality theory was used as an organising principle. Interviews were transcribed verbatim and analysed using a thematic framework analysis.

**Results:**

For BMSM with heterogeneous social groups, discussions about sexual health were challenging because of the intersection of ethnic background, family history and religion. This limited conversations about PrEP to gay male friends who often held stigmatising views of condomless anal intercourse. BMSM reported exclusion from gay male spaces (online and offline) which could serve to restrict exposure to PrEP messages. Stereotypes of BMSM intersected with negative conceptions of PrEP users, limiting acknowledgement of PrEP candidacy. For those who had attempted to or successfully accessed it, PrEP was framed as a strategy to mitigate risk and to guard against further stigma associated with HIV infection.

**Discussion:**

BMSM operate within a complex set of circumstances related to the intersection of their sexual, ethnic, cultural and religious identities, which shape PrEP acceptability. Interventions which seek to facilitate uptake in this group must be attentive to these. Health promotion and clinical services could seek to facilitate nuanced discussions about the merits of PrEP for those at frequent risk, perhaps while also providing publicly visible PrEP role models for BMSM and other marginalised groups.

## Background

Since 2015, HIV incidence in men who have sex with men (MSM) in England has undergone a remarkable shift with steep falls observed in London and some other metropolitan areas.^1 2^ In anticipation of further declines in these areas, and in others, it is important we carefully attend to the ethnicity of those continuing to be diagnosed with HIV, lest we exacerbate existing inequality. Over the course of the HIV epidemic in the UK, black MSM (BMSM) have historically had both higher HIV prevalence and incidence, despite similar levels of risk compared with other groups.^3–5^ HIV incidence in an English national cohort of clinic attending BMSM was 3.2 per 100 person-years, compared with 2.0 for all MSM.^3^ In a cross-sectional study, 2.8% of BMSM were diagnosed with HIV in the preceding 12 months compared with 1.1% of MSM overall.^4^ Although long a priority group in the USA, intensive and focused HIV prevention interventions for UK BMSM, who make up 5% of clinic attending MSM,^6^ have not been a reality. HIV incidence among English MSM (and in the UK as a whole) peaks between the ages of 18 and 45, indicating this subgroup may be especially appropriate for intervention.^7^

In recent years attention has focused increasingly on HIV pre-exposure prophylaxis (PrEP), where an HIV negative individual takes antiretroviral medication before exposure to prevent transmission. PrEP has been demonstrated to be highly effective at preventing HIV.^8–11^ PrEP may be a useful tool to address HIV health inequalities, especially if targeted towards groups with the highest HIV burden. In the USA, however, where PrEP is more widely available although through a vastly different health system, PrEP outcomes tend to be worse among BMSM.^12 13^

In England, PrEP was first made available to 550 men through the PROUD (Pre-exposure option for reducing HIV in the UK: an open-label randomisation to immediate or deferred daily Truvada for HIV-negative gay men), a pragmatic open-label randomised controlled trial (RCT) beginning in 2013.^8 14^ Although PROUD reported a high level of effectiveness^8^ there have been significant structural and policy-related challenges to making PrEP available on the England National Health Service (NHSE). Although the NHSE has now commissioned IMPACT (PrEP Impact Trial), an implementation study which provides PrEP to 10,000 participants from October 2017 (with a planned extension to 26,000 places), there was a gap in provision for a considerable time. It was within this gap, and as a consequence of PrEP only being made available to 10,000 participants, that some MSM sought to access it via alternative means. Self-sourcing generic versions of PrEP medication online was the predominant method for access, although numbers of individuals accessing PrEP in this way are unclear.^15^ PrEP is also currently available through a non-inferiority RCT comparing Truvada (tenofovir and emtricitabine) to a new agent with uncertain efficacy.

Motivation for, and barriers to, using PrEP have been intensively studied. Frequently cited motivators include: high-risk perception which men hope PrEP will mitigate; increased pleasure associated with condomless sex; and desire to avoid HIV infection.^16–20^ Reported barriers include prohibitive cost; lack of risk perception; unacceptable clinical services; concern about side effects; and issues of medicalisation and stigma.^16–22^ Our first paper published from this study examines PrEP health service preferences. We found BMSM preferred convenient clinic locations outside their home communities, and felt the skills of clinical staff were especially important in mediating access to potential PrEP services (see ref 22).

Mindful that PrEP-related interventions ought not to further exacerbate existing health inequality, as may be the case in the USA, this study sought to understand the motivations and barriers to PrEP uptake among BMSM in London. This was achieved by exploring the potential role of peer and group norms in personal understandings of, and decision-making processes surrounding, PrEP use and perceptions of PrEP candidacy.

It should be noted at the point of data collection PrEP was available to a small number of individuals through the PROUD study but wider access free of charge was not a reality in London. Individuals had begun to self-import generic PrEP formulations from abroad, but this practice was not yet widespread.

## Methods

Twenty-five BMSM took part in in-depth interviews between April and August 2016. Intersectionality theory provided an organising principle and helped frame study conceptualisation, data generation, analysis and write-up. This theory emphasises the interactive effects ethnicity, sexual orientation, class, language and other personal characteristics have with each other, profoundly shaping life experience and healthcare seeking.^22–24^ A full account of our methods can be found elsewhere.^22^

Participants were recruited through adverts on geolocation social networking applications (apps) (Growlr and Scruff), through social media (Facebook and Twitter) and through a mailing list for the PROUD study. Individuals provided demographic details (age, gender, sexual orientation, HIV testing history and number of condomless anal intercourse (CAI) partners) on an online survey, and if eligible, their contact information and consent for follow-up from the study team. Potential participants were also asked whether they had heard about PrEP; knew someone who had taken PrEP; had tried unsuccessfully to access PrEP; or had accessed PrEP. We sought to ensure diversity across these characteristics.

Eligible participants described their ethnicity as black or mixed race using standard UK ethnicity codes, were between the ages of 18 and 45 and reported sexual behaviour consistent with clinical definition of PrEP candidacy: at least one instance of CAI with a man the preceding 3 months. Participants were compensated £40.

All interviews were face to face in the offices of the lead author’s host institution and were conducted by one of the three authors. A semistructured topic guide explored: PrEP knowledge; sexual history and risk; social contact and peers; and health service engagement. The analysis presented in this paper relates to the first three of these topics. Interviews were recorded and transcribed verbatim.

A thematic framework analysis was developed fusing the approaches outlined by Clarke and Braun, and Ritchie and Spencer.^25 26^ This approach involved following steps 1 through 3 in thematic analysis to develop a framework which was then used for analysis. We first read transcripts closely and extracted themes, being mindful of the intersectional effects of identity. These were arranged into groups, with meta-themes emerging above them. This framework was piloted by two authors, refined and applied to all transcripts. Each author was responsible for some analysis and met to discuss and refine the framework periodically in order to ensure accuracy. In this framework analysis, those who had not attempted or used PrEP were classified as ‘PrEP naïve’, and those who had used PrEP as ‘PrEP experienced’. Those who had unsuccessfully attempted to access PrEP were classified as ‘attempted to access’.

## Results

Sixty-three BMSM filled in the recruitment survey, of these 24 were ineligible and 14 either declined or were not selected for interview. In the final sample more (n=14) described their ethnic background as Black African compared with those of other black ethnicities. The majority were highly educated and engaged with sexual health clinics. See table 1 for full sample details.

**Table 1:** Participant demographics

Attribute	Options	n
Age (mean 31.1)	18–25	8
	26–35	10
	36–45	7
Ethnicity	Black/Black Caribbean	7
	Black/Black African	14
	Black/Black British other	4
Sexual orientation	Gay/homosexual	19
	Bisexual	5
	Other	1
Education	Low*	1
	Medium†	9
	High‡	15
Last HIV test	>3 months	18
	>12 months	4
	<12 months	3
PrEP experience	PrEP naïve	17
	Unable to access PrEP	3
	PrEP experienced	5

*General Certificate of Secondary Education (GCSE) and below.

†A-levels or equivalent, higher education below degree level.

‡Degree or higher.

PrEP, pre-exposure prophylaxis.

Many of the motivations (e.g. efficacy, affordability, preference for CAI) and barriers (e.g. cost, lack of risk perception) identified in our analysis are common across groups of MSM in other settings.^27^ For this manuscript, we focus on issues which are especially salient for BMSM given the necessity of engaging this group. Three main themes related to motivations or barrier to using PrEP emerged in our analysis, which are discussed in turn.

### PrEP-related knowledge, support and discussion

Discussions about sexual health and PrEP were challenging for individuals with heterogeneous social groups because of the intersection of ethnic background, family history and religion. This was most pronounced among those who were not widely open about their sexual practice and attraction with their social networks, but also for many BMSM who described widespread acceptance of their sexual orientation. This was also most strongly reported among BMSM from African backgrounds. These social dynamics limited conversation about sex mainly to other gay or bisexual men who became the predominant source of sexual health information and support.

For the most part it [conversations about sex] doesn’t really come up. But just for a, I say handful but I mean one or two, then maybe what happened or whether you were with this person last night. Those sorts of things. But just from my background, it’s kind of a prudish background, but you never really talk openly about sex. (22-year-old bisexual man, Black British African, PrEP naïve)

Discussions about CAI with other gay and bisexual men were, however, often very challenging due to stigma around so-called ‘barebacking’. This stigma, which was most pronounced in the PrEP naïve, significantly limited PrEP discussions. These conversations often framed PrEP as irresponsible, and a tool to facilitate deviant behaviour.

Those who were PrEP experienced or had tried unsuccessfully to access PrEP described these conversations as being more straightforward. These men often spoke to their friends and peers about sex and PrEP, although largely with select individuals or in small groups. Some felt, however, that should they be able to access PrEP, they would discuss it very widely in an effort to increase awareness:

*If you took PrEP do you think you would tell your friends?*

Oh yeah absolutely, yeah and if I took PrEP and I knew how to get PrEP I would literally advertise to as many people as I want. In all honesty I think it is absolute outrageous that PrEP is not available by now. (23-year-old gay man, Black British African, unable to access PrEP)

This underlines a dynamic where a relatively small number of men are having conversations about PrEP, and with men whom they consider will not have opposing opinions and views. This limits the potential for peer-to-peer health promotion discussions beyond networks of men who could benefit from understanding the opportunities of PrEP, directly from men who are using it.

### Participation, marginalisation and exclusion

Participating in gay male spaces, both online and offline, was often a fraught experience for BMSM, limiting exposure to health promotion initiatives, including those disseminating PrEP information. This was related to inadequate representation of black men in the spaces themselves, as well as experiences of racism ranging from the subtle to the profound. A lack of representation of images of black men in gay culture also limited feelings of belonging for some, as whiteness was felt to be prized within these spaces. This lack of focus could make accessing the scene difficult for some groups.

If I go to the gay scene, I don’t really see representation of black or African men. That’s a big reason [for not going]. In that sense as well, I feel like I don’t fit in there. I feel like I’m a minority within another minority. (25-year-old gay man, Black British Caribbean, PrEP naïve)

In addition to a lack of representation, all men interviewed described negative, racially charged encounters ranging from mildly annoying to profoundly upsetting when inhabiting gay male spaces.

So I’m a little bit hardened to anything else, but it can get a bit annoying. You know, being told things like: ‘You’re good looking for a black guy.’ I’m like: ‘You don’t realise how racist that is!’ [Laughs] Just say: ‘You’re good looking’ and leave it at that. (36-year-old gay man, Black British Caribbean, PrEP naïve)

This was rarely identified as racism as it was not characterised as being ‘aggressive’ or ‘violent’. Rather it functioned to limit participation and one’s feelings of value. This exclusion had dual implications. First, these barriers to accessing the scene function to limit exposure to health promotion messages through exclusion, as gay spaces have been a key domain of dissemination. It also reinforced particular social dynamics valuing only certain heavily racialised traits of black men.

### Stereotypes, sexualisation and responsibility

Harmful beliefs and stereotypes of black men intersected with those of PrEP users, limiting feelings of PrEP candidacy and therefore PrEP acceptability. Conversely, for the PrEP experienced and those who had attempted to access PrEP, this same marginalisation was also a major motivator for access.

Black men frequently described encountering stereotypes of BMSM, which had impacted their perceived position within the gay sexual sphere. Belonging was heavily predicated on ideals of black male sexuality alongside stereotypes of muscled bodies, large penises, promiscuity and sexual dominance. These narratives were universally contested and largely thought of as harmful, especially for those who did not fit expectations held by other gay men.

It’s like anything where you’re an ethnic minority, the majority pushes one type of beautiful, or one type of ideal. And I think that not being that ideal can be quite annoying sometimes, because then you’re having to, not fight, but you’re, kind of, having to work a little bit more. I find it doubly annoying because on the gay scene black guys have a certain stereotype, and I’m just, like, that’s really boring. (37-year-old gay man, Black British Caribbean, PrEP naïve)

PrEP use was also thought about in highly idealised terms by many. These stereotypes mirrored and intersected with stereotypes of black men, limiting acknowledgement of PrEP candidacy. Potential PrEP users were often seen as deviant, out of control and reckless. These narratives were drawn from accounts of PrEP users disseminated through social media, and in juxtaposition with existing norms about condom use and numbers of partners. PrEP-naïve BMSM struggled to identify themselves within these narratives, limiting perceptions of their own PrEP eligibility.

It has to do with the publication for it. It’s seems to be something you take when you engage in very illicit non-safe sex activity with multiple men, which isn’t something that I tend to do. Hence the reason I don’t do PrEP. (31-year-old gay man, Black British African, PrEP naïve)

This idea that taking PrEP marked oneself as especially promiscuous intersected with racialised stereotypes which deemed black men to be hypersexual. PrEP use therefore meant taking on an additional, stigmatised identity for one already doubly marginalised as black and gay. Although these two issues were rarely connected by BMSM themselves, this was reflected by a mirroring of language between these two themes which emerged during analysis; the negative stereotypes of PrEP use related especially to promiscuity and hypersexualisation were very similar to harmful stereotypes of BMSM described in interviews.

Conversely, for the minority who had accessed or attempted to access PrEP, this was framed in terms of accepting and mitigating natural risk in a responsible way.

Even if you're in a monogamous relationship. I mean—you're monogamous, you don't know what your partner's doing. And then you catch some shit, what are you doing to do? […] That's why PrEP for me is about control. I'm on control of my own health. I'm choosing to protect myself from HIV. (28-year-old gay man, Black British African, PrEP user)

Further, the knowledge that being black and gay put them at higher HIV risk meant PrEP was protective against a different—but highly stigmatised—identity: that of a black, gay, HIV-positive man.

[…] I remember a black fella telling me it is one thing to be a gay black man on Grindr especially in the world of no fats, no fems, no Asians, no blacks for example but if you then add HIV on the top of that then you are even pushed lower down the hierarchy of who is desirable and who is not desirable. (23-year-old gay man, Black British African, unable to access PrEP)

## Discussion

Our study identified three main themes related to PrEP acceptability among BMSM in London. First, BMSM who had very mixed social groups in terms of sexual orientation and ethnicity tended to report limiting discussions about sexual health to their gay male friends. Although other gay men were often a source of support and sexual health information, open and honest discussion about CAI was challenging, thereby limiting conversation about PrEP. Second, participation in gay male spaces for BMSM online and offline was limited by a lack of representation and occasional hostile attitudes. This potentially served to reduce exposure to health promotion activities through physical, social and affiliative exclusion which limited participation. Finally, stereotypical understandings of PrEP use intersected with racist ideals of BMSM’s sexuality, limiting self-identification as a potential PrEP user. Conversely, concerns about further marginalisation were a motivator for some BMSM to take PrEP as a strategy to guard against further stigma.

Our findings detail a complex set of circumstances that BMSM operate within, which may function as a barrier to PrEP uptake. This poses distinct challenges for health promotion and other PrEP dissemination activities that seek to reduce health inequalities. The continued stigma surrounding CAI and its association with PrEP use will be a key issue for all groups of MSM. For BMSM, however, this appears to pose additional challenges because of cultural attitudes surrounding discussions of sex and sexuality. Health promotion activities must seek to engage with this issue sensitively and pragmatically by facilitating nuanced discussion about the benefits of PrEP for those at frequent risk. Given the strong association of PrEP use with ‘deviant behaviour’ and the lack of discussion among BMSM about the benefits of PrEP, an appropriate response may be to provide publicly visible PrEP role models or ambassadors which could seek to challenge these harmful narratives.

Given the challenges BMSM face, PrEP health promotion activities for this group will have to move beyond traditional approaches that rely on access to gay male spaces, both physical and online. Instead, a more targeted strategy could be appropriate, perhaps training BMSM PrEP navigators who can work in clinical settings helping to identify potential PrEP beneficiaries and providing appropriate information and support. This approach has also been recommended in the USA^28^ and aligns with our previous publication which detailed how BMSM are often uncomfortable accessing services from those of similar backgrounds to themselves, but engagement with healthcare workers of a similar ethnicity who are perceived to be MSM can be an empowering experience.^22^ This publication also recommends enhanced training for healthcare workers to more effectively engage BMSM.^22^

The experiences of racism described by our participants were troubling. It is crucial all organisations involved in health promotion and advocacy initiatives with MSM incorporate challenging racism into their routine and ongoing programmes. Initiatives to reverse the health inequalities seen in this group will have limited success in a context of structural marginalisation.

### Strengths and limitations

This is the first research to explore motivations and barriers to PrEP use among this key population in the UK. The use of intersectionality theory enabled us to examine the critical interconnectedness of social roles that our participants inhabit in ways that other theories might not facilitate.^23 24^

However, despite repeated efforts, we struggled to engage BMSM with low educational qualifications and those who did not access health services, groups with acknowledged HIV prevention need.^29^ The barriers we describe could therefore be different (or perhaps more pronounced) in those groups. Barriers and facilitators for BMSM will also likely differ by geographical context, meaning these may not be representative outside of London. In addition, participants were recruited largely from apps and social media, meaning those who do not use these platforms are not represented in our sample. Future research should attend to these limitations and examine whether men outside of London (a large gay urban centre) and less active on social media (which may provide an avenue to enhanced PrEP awareness) face similar barriers to access and uptake of PrEP.

Mindful that the standard black ethnicity categories used in the UK can mask considerable heterogeneity, we also recommend future research is attentive to the diversity of Black African and Black Caribbean communities, including recent migrants and those with longer roots in the UK.

## Conclusion

Sexual health discussions for BMSM were often constrained to like-minded friends and peers, limiting the potential for peer-to-peer learning about PrEP. Exposure to health promotion messages in this group may be limited by exclusion from gay male spaces. Marginalisation intersects with negative stereotypes of PrEP candidates, limiting acceptability for some and ultimately impacting the potential for this new technology to equitably benefit a population of MSM in the UK who are most at risk of acquiring HIV.

**Key messages**

- PrEP discussions are limited to other gay male friends for BMSM with very mixed social groups.
- BMSM frequently report exclusion from online and offline spaces, limiting exposure to health promotion messages about PrEP.
- Stereotypes of BMSM intersect with negative conceptions of PrEP users which limits acknowledgement of PrEP candidacy in this group.
- PrEP use can also be understood to be a form of control over risk, potentially guarding against further stigma.
